# The Influence of 2,3,7,8-Tetrachlorodibenzo-*p*-dioxin (TCDD) on Hematological Parameters During Experimentally Induced Pleuritis in Rats

**DOI:** 10.1007/s10753-012-9558-y

**Published:** 2012-10-26

**Authors:** Ireneusz Całkosiński, Joanna Rosińczuk-Tonderys, Justyna Bazan, Katarzyna Dzierzba, Monika Całkosińska, Jacek Majda, Maciej Dobrzyński, Agnieszka Bronowicka-Szydełko

**Affiliations:** 1 Department of Nervous System Diseases, The Faculty of Health Science, Wroclaw Medical University, Bartla 5, 51-618 Wroclaw, Poland; 2 Department of Medical Biochemistry, Wroclaw Medical University, Chalubinskiego 10, 50-368 Wroclaw, Poland; 3 Outpatient Clinic Medcom in Wojkowice, Wojkowice 28B, 55-020 Zurawina, Poland; 4 Department of Diagnostics Laboratory, 4th Military Academic Hospital in Wroclaw, Weigla 5, 53-114 Wroclaw, Poland; 5 Department of Conservative Dentistry and Pedodontics, Wroclaw Medical University, Krakowska 26, 50-425 Wroclaw, Poland

**Keywords:** Dioxins, TCDD, inflammatory reaction, acute phase, hematological parameters

## Abstract

Proper functioning of homeostatic mechanisms is characteristic for every healthy organism and enables adapting to environmental changes. These complicated systematic reactions can neutralize the harmful stress factors leading to various inflammatory reactions. The aim of this study was to determine dynamic changes in the inflammatory reaction after single 2,3,7,8-tetrachlorodibenzo-*p*-dioxin (TCDD) administration of 5 μg/kg body weight into rats with experimentally induced pleuritis. These changes were observed by monitoring the hematological blood parameters during inflammation. The obtained results proved that dioxins contribute to various changes in the character of the inflammatory response. TCDD administration before pleuritis initiation caused an increase of lymphocytes and significant decrease of the number of neutrophils during inflammation. The current study proved that administration of low TCDD dose (seven times lower than used in other studies) can cause thymus, spleen, or lymphatic gland atrophy. This finding indicates the toxic influence of small TCDD dose especially on the immune system.

## INTRODUCTION

The inflammatory reaction is caused by several physical factors (*e.g.*, ionizing radiation, magnetic field, ultrasounds), chemical factors (*e.g*., carrageenan, acids, bases, dioxins), and biological factors (*e.g.*, bacteria, viruses, fungi, protozoa, exo- and endotoxins). These agents are able to cause disorder of the local homeostasis. The defensive reaction stimulates several processes to restore the original state [1, 2]. Dioxins are reactive compounds which stimulate COX-2 and have pro-inflammatory properties—they induce inflammation of the skin called chloracne syndrome. Dioxins can influence different inflammation phases in an organism which can be checked by monitoring biochemical or hematological blood parameters [3].

**Fig. 1:**
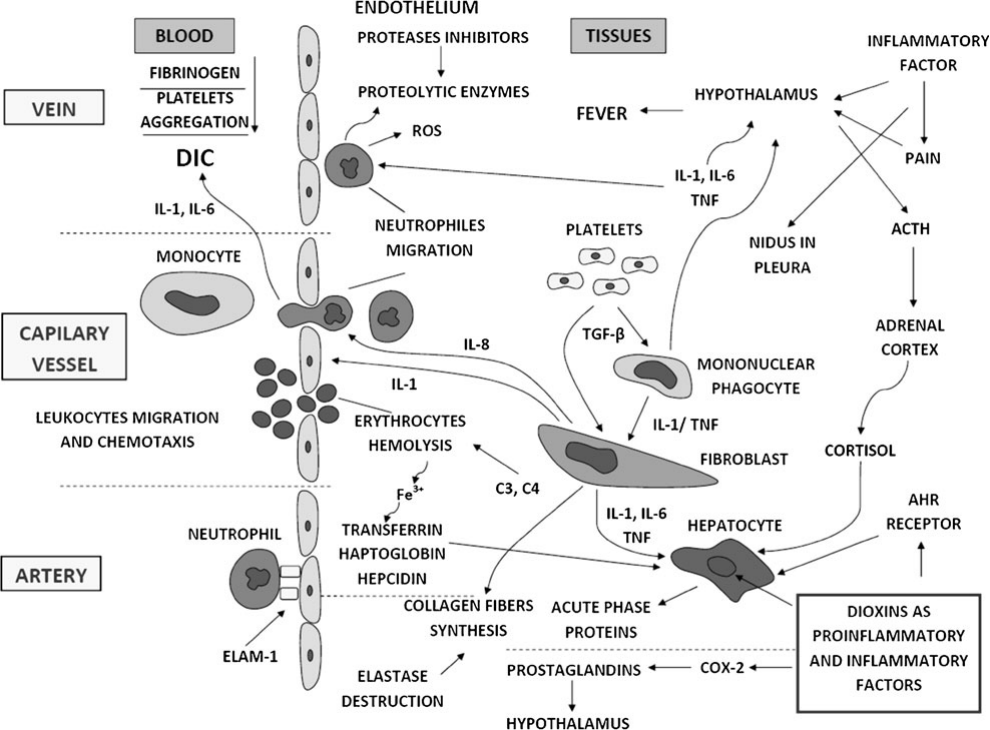
The schematic representation of the cell-mediated immune and humoral response during the inflammatory reaction course. Modified from [3].

The inflammatory response has a long-lasting and multistage characteristic, and its specific dynamics are dependent on the phase reaction [4–6]. Cell mobility, *e.g.*, migration, adhesion, diapedesis, chemotaxis, and humoral immune response, is often observed [7]. In other cases, there are inflammatory mediators locally occurring in humor (*e.g.*, histamine, CRP protein, complement proteins, interleukins, prostacyclins, prostaglandins, thromboxane) [8–10]. The homeostatic response is also characteristic and the symptoms are platelet aggregation, blood clotting, and disseminated intravascular coagulation (DIC) [11, 12]. Hematological changes observed in the inflammatory reaction are closely associated with activation of the C3 and C4 complement system (Fig. 1).

The local inflammatory changes sometimes cause erythrocyte hemolysis, platelet aggregation, and adhesion to the ablated epithelium cells of capillary vessels [7]. They are responsible not only for the micro-blood clot formation but also for the occurrence of the DIC [4, 13]. These processes are dynamic and rapid. The rise of the permeability of vessels causes the swelling of the connective tissue structures, *e.g.*, collagen fibers and interstitium of connective tissue [14]. The fibroblasts are induced to divide and the blood corpuscles such as collagen and, as a consequence—elastic fibers—are produced. They form the demarcation line of the inflammation and neutralize the toxic effect of inflammatory products [4, 15]. The slow blood flow in the inflammatory focus causes an increase of the vascular resistance and makes the diapedesis of leukocytes easier (*e.g.*, micro- and macrophages) [16, 17]. Consequently, the inflammatory mediators are absorbed on their cell membranes and the increase of the permeability of blood vessels leads to serum protein migration through this barrier (*e.g.*, fibrinogen). After the intravascular coagulation, fibrinogen forms a local network where cell elements fall [18]. The intracellular fluids flow across the lymphatic vessels to regional lymph nodes in the inflammatory focuses. Swelling and pain are the most characteristic features [19, 20]. A lot of neutrophils, eosinophiles, and thrombocytes appear in the fourth and fifth hour of the inflammatory reaction [19, 21, 22]. The leukocytes produce free radicals and superoxide ions [20, 23–25], and granulocytes activate the release of prostaglandins (*e.g.*, PGE_2_) [10, 14, 19], which accelerate the effects of bradykinin, histamine, and serotonin [9, 26–28]. Migrating granulocytes in the inflammatory focus cause the local necrobiotic changes, and they damage the intrafollicular and intralobular septum in the lungs because of the presence of elastase in neutrophils [4, 6, 21, 22, 28].

Currently, no data has shown dioxin interaction on the basic morphologic blood parameters including the erythrocyte and leukocyte system, which is used for inflammation monitoring. Probably, immunosuppressive dioxin action can significantly influence blood parameters, mainly by generation of free radicals and induction of pro-inflammatory cytokines. Dioxins, by interaction on the cell receptor (aryl hydrocarbon receptor—AhR), contribute to induction of some cytokine formations responsible for the development of some blood cells. Furthermore, TCDD—the agonist of this receptor—inhibits the expression of mRNA of IL-6 in the presence of lipopolysaccharide (LPS) and interacts with hematopoietic cells and lymphocytes B [29]. Studies carried out by Rodriguez-Sosa *et al.* have shown that TCDD added to lymphocytes B breeding stimulated by LPS and IL-4 causes the inhibition of secretion of some immunoglobulins: IgG1, IgE, and IgM [30]. Moreover, clinical studies have proven that TCDD present in the milk of polar bear females is responsible for the immunity decrease in their offspring [31]. Dioxins cause long-lasting immunosuppression of pre-lymphocytes B in marrow which is connected with induction of apoptosis processes. The AhR activation, as a result of small dioxin doses, influences hematopoiesis of immature lymphocytes [32]. Studies on monkeys have shown a decrease of complete and relative amount of lymphocytes according to the main leukocyte number during 3 weeks from TCDD application of 300 ng/kg body weight (b.w.). These studies have also pointed to a 20 % decrease of CD4 lymphocytes [33, 34]. Studies on mice immunized by SRBC which were treated with 5 μg/kg b.w. of TCDD have shown decrease of CD4 and CD8 lymphocytes in relation to the control group in which these numbers have increased [35].

## MATERIAL AND METHODS

### Experimental Animals

Female rats from the *Buffalo* inbreeding strain (body mass 130–150 g, age 9–11 weeks) were used in this study [36]. The age and body mass parameters of these animals had to be very similar because the reactivity of some inflammatory factors depends on individual features, such as age, sex, or strain (under invariable environmental factors) [37]. The rats were bred from the Department of Pathomorphology in Wroclaw Medical University. All the rats were kept under the same conditions: they were kept in polystyrene cages (60 cm × 40 cm × 40 cm) with metal lids (six animals in each cage). The experiments were carried out in air-conditioned rooms—the temperature oscillated between 21 and 22 °C and the humidity of air was 62–63 %. Rats were maintained in a light/dark cycle for 12/12 h. The rats were fed by the standard diet “Murigran” and received water *ad libitum* [36]. All experiments with the use of rats were permitted by The Local Bioethics Council for Animal Experiments (permission number: 23/2001).

**Fig. 2:**
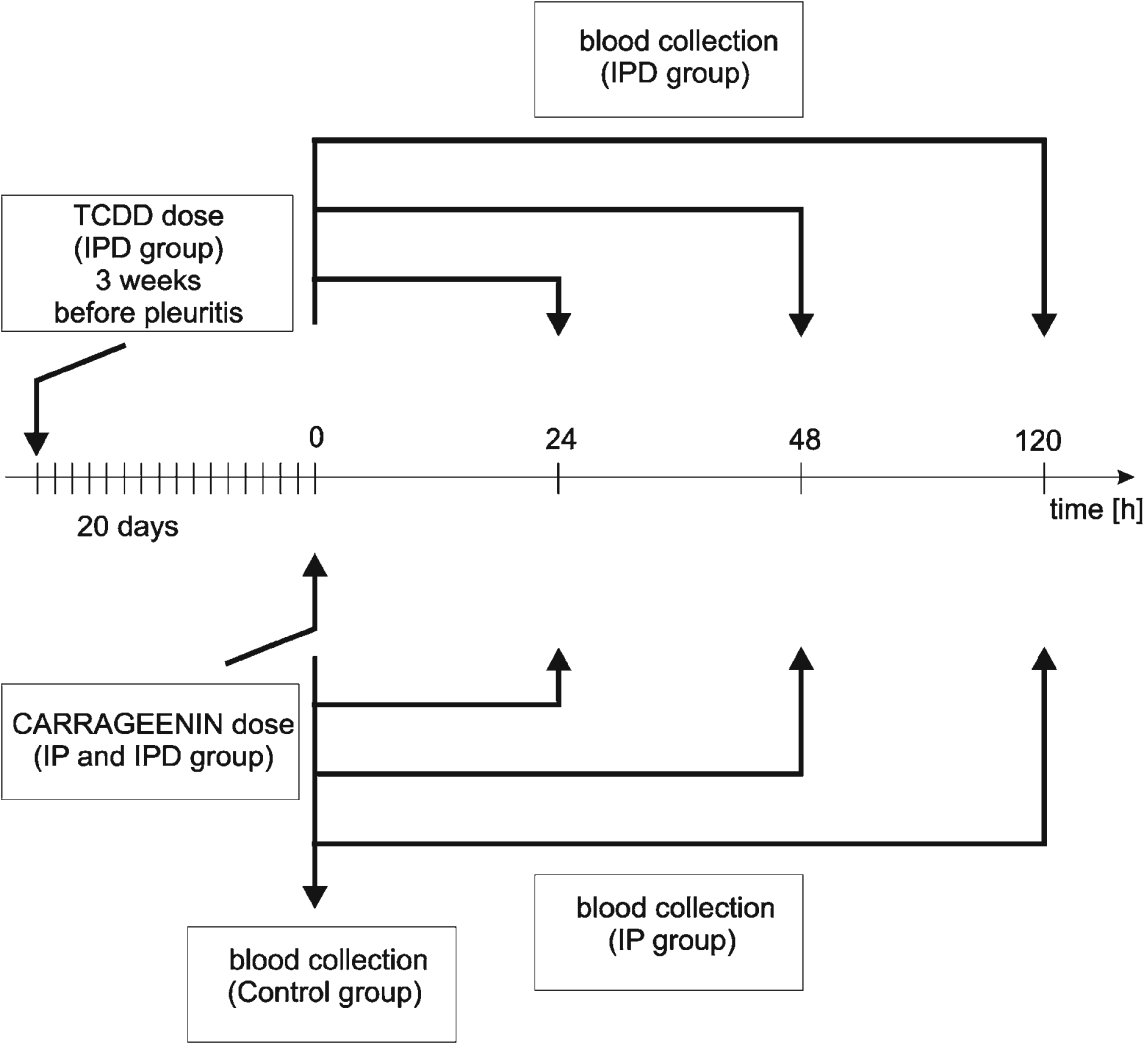
The scheme of the induction of the pleuritis in rats with temporal monitoring of the biochemical parameters of inflammation reaction after TCDD administration (IP—group of rats with induced pleuritis, IPD—TCDD-dosed group of rats with induced pleuritis after 20 days, Control—control group of animals without induced pleuritis (not shown).

TCDD powder (Sigma-Aldrich, Poland) dissolved in DMSO was applied in a dose of 5 μg/kg b.w. (intramuscularly in a volume of 0.7–0.8 mL) [3]. Pleuritis was induced by a single dose of 1 % carrageenan solution in a volume of 0.15 mL intrapleural. Carrageenan (Sigma-Aldrich, USA) extracted from *Chondrus chrispus algea* had been dissolved before the experiments in 0.9 % NaCl solution (Polfa, Poland). Next, this solution was injected into the intrapleural cavity (in a volume of 0.15 mL) at four to five intercostal spaces on the right side. Prior to blood collection, rats were under anesthesia induced by pentobarbital (Biochemie GmbH) in a dose of 30 mg/kg b.w. administered intramuscularly (Fig. 2). In order to avoid hemolysis and enzyme appearance, characteristic of damages tissues, blood was drawn from the aorta by catheterization in a volume of 4–5 mL.

Rats were divided into three groups:

1. Control—The control group of 30 female rats without inflammation (intact group); physiological group without carrageenan and TCDD applications. The blood was collected in the 120th hour of the experiment (Fig. 2).
2. IP Group—A group of 60 female rats with pleuritis induced by a single intrapleural injection of 0.15 mL of 1 % carrageenan solution (Sigma-Aldrich) administered in the first minute of the experiment (Fig. 2). The blood was collected at three time points at the 24th (*n* = 20), 72nd (*n* = 20), and 120th hour (*n* = 20) after carrageenan injection (Fig. 2).
3. IPD Group—A group of 60 female rats were injected intramuscularly with a single dose of TCDD (5 μg/L kg b.w.) on the 20th day before 1 % carrageenan application. A single dose of 0.15 mL of 1 % carrageenan solution was applied intrapleurally to these animals (Sigma-Aldrich) on the 20th day after TCDD application—in the first day of the experiment (Fig. 2). The blood was collected at three time points at the 24th (*n* = 20), 72nd (*n* = 20), and 120th hour (*n* = 20) after carrageenan injection (Fig. 2).

### Marking of the Types of Hematological Parameters During Experiments

The process of inflammation in rats treated with carrageenan and TCDD was monitored by valuation and comparison of the following hematological parameters: erythrocytes (RBC), hemoglobin (HGB), hematocrit (HCT), red blood cell distribution width (RDW), mean corpuscular volume (MCV), mean corpuscular hemoglobin (MCH), mean corpuscular hemoglobin concentration (MCHC), platelets (PLT), mean platelet volume (MPV), thrombocrit (PCT), platelet distribution width (PDW), leukocytes (WBC), neutrophils (NE), lymphocytes (LY), monocytes (MO), eosinophiles (EO), and basophiles (BA). The basic hematological parameters were marked using standard diagnostic tests and the Sysmex XT-1800i hematological analyzer (Sysmex Poland Ltd.) at the Diagnostic Laboratory of the 4th Military Academic Hospital in Wroclaw, Poland.

### Statistical Analysis

The hematological parameter values in rat blood were analyzed using Statistica 9.0 (StatSoft Ltd). The data obtained were presented as arithmetic means of parameters (*X*). For the determined number of animals used in the experiment, standard deviation (*D*) and the ranges of minimal (MIN) and maximum (MAX) values of parameters were also calculated. Data distribution was tested using the Kolmogorov–Smirnov normality test and particular groups were compared using Student’s *t* test, taking Bonferroni correction under consideration to determine levels of significance (*P*). Data were divided into three groups and indicated as follows: *—0.05 ≥ *P* > 0.01; **—0.01 ≥ *P* > 0.001; ***—0.001 ≥ *P*; and NS—not significant. Correlation analysis was conducted with Pearson (*r*) correlation test. Indicators for correlation are as follows: *r* = 0, the variables are not correlated; 0 < *r* < 0.1, dim correlation; 0.1 ≤ *r* < 0.3, weak correlation; 0.3 ≤ *r* < 0.5, average correlation; 0.5 ≤ *r* < 0.7, high correlation; 0.7 ≤ *r* < 0.9, very high correlation; and 0.9 ≤ *r* < 1, almost total correlation. Statistical data are collected in tables and presented on diagrams.

## RESULTS

### Analysis of Hematological Parameters Obtained in Experimentally Induced Pleuritis After TCDD Administration in Rat

#### Erythrocytes (RBC)

**Fig. 3:**
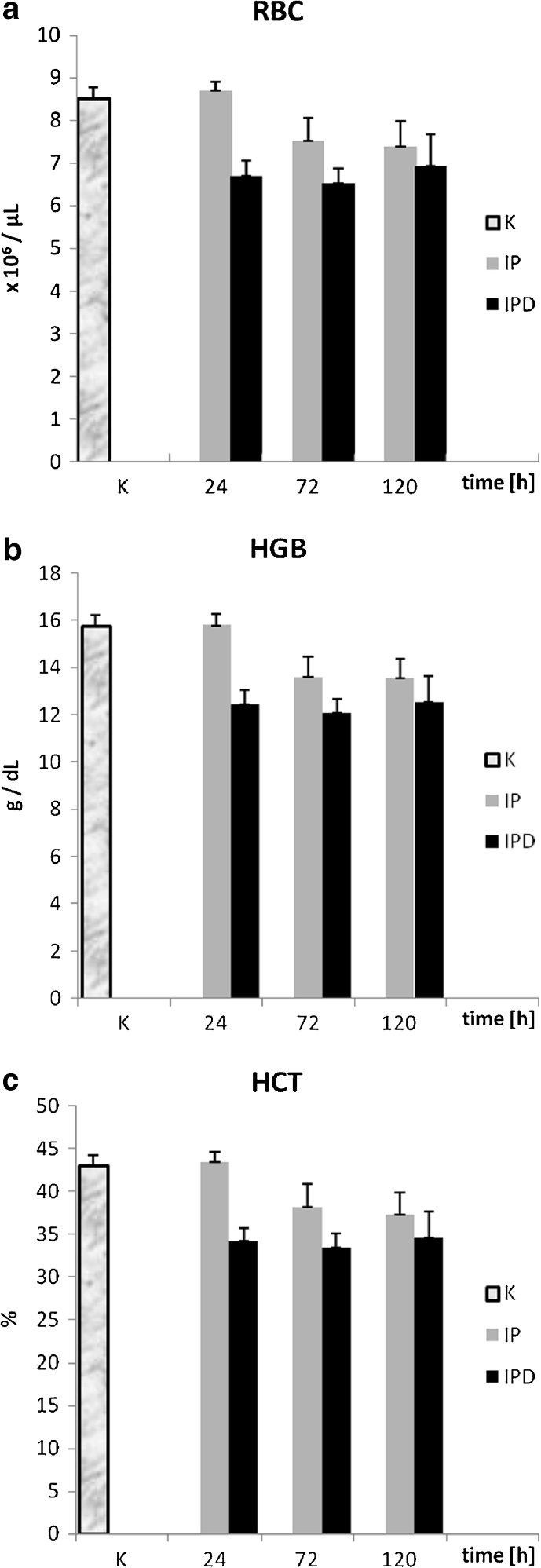
**a** The influence of TCDD on erythrocyte concentration during the experimentally induced pleuritis in rats. **b** The influence of TCDD on hemoglobin concentration during the experimentally induced pleuritis in rats. **c** The influence of TCDD on hematocrit parameter during the experimentally induced pleuritis in rats.

**Fig. 4:**
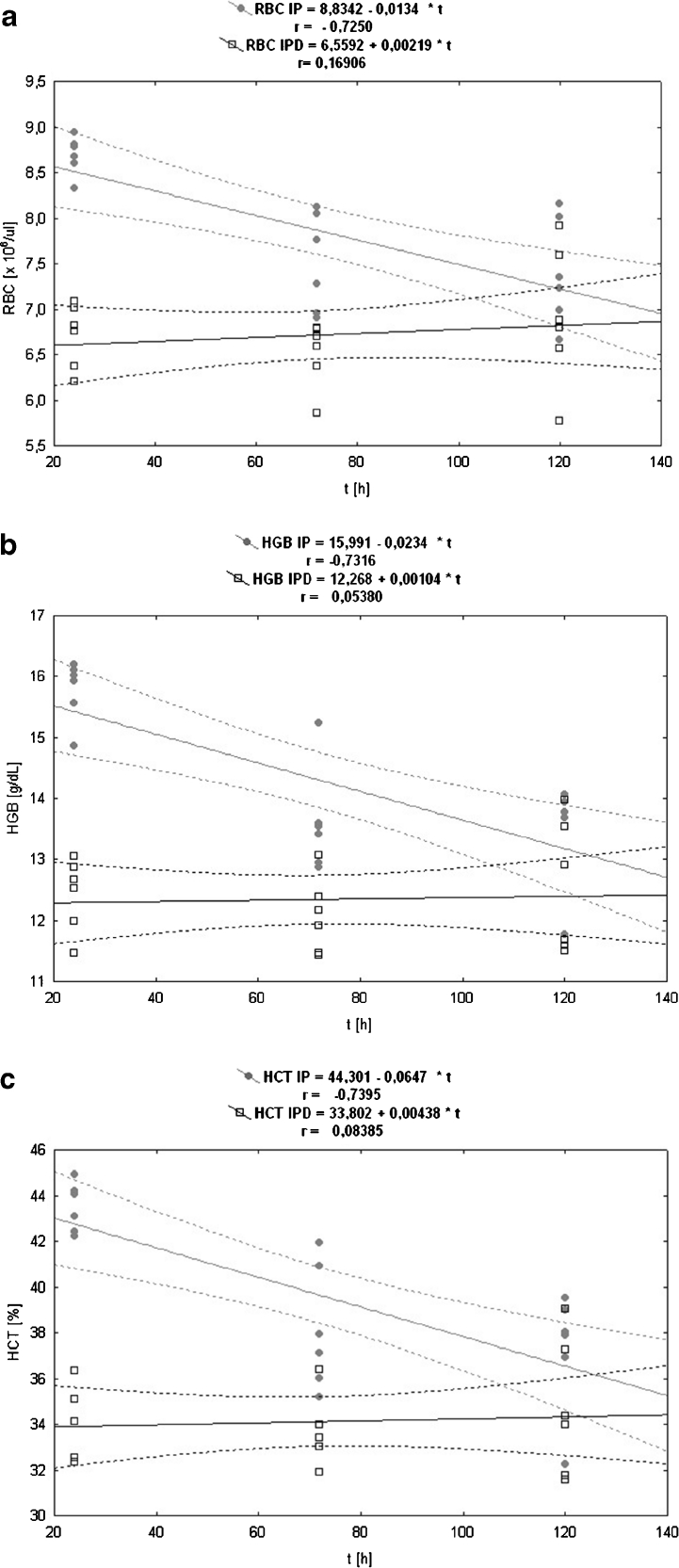
**a** The linear regression of the influence of experimentally induced pleuritis (IP) and dioxin exposition (IPD) on the erythrocyte (RBC) parameter in rats. **b** The linear regression of the influence of experimentally induced pleuritis (IP) and dioxin exposition (IPD) on the hemoglobin (HGB) parameter in rats. **c** The linear regression of the influence of experimentally induced pleuritis (IP) and dioxin exposition (IPD) on the hematocrit (HCT) parameter in rats.

The RBC level for the IP group in relation to the control group is lower during the total experimental time (except the first blood measurement at the 24th hour in which the RBC level for the IP group is higher than in the control group) and the RBC value for the IP group is still dropping within 120 h of the experiment (Fig. 3a). The RBC number in the IPD group is lower than in the IP group at all three measurement points, and the differences of RBC concentration between the IP and the IPD group are statistically significant at the 24th and 72nd hour of pleuritis (Table 1). The correlation of RBC number in relation to time in the induced pleuritis group (IP) is negative (Fig. 4a), and its value is very high and statistically significant (*P* = 0.001). In contrast, the correlation in the induced pleuritis group after dioxin injection (IPD) is positive, weak, and statistically insignificant (Table 2).

**Table 1:** Values of Erythrocyte and Platelet Blood Parameters Received at the 24th, 72nd, and 120th Hour of the Experiment

		RBC (×10^6^/μL)	HGB (g/dL)	HCT (%)	MCV (fL)	MCH (pg)	MCHC (g/dL)	PLT (×10^3^/μL)	MPV (fL)	PCT (%)	PDW (%)
K	*N*	14	14	14	14	14	14	14	14	14	14
	*X*	8.52	15.74	43.02	50.49	18.11	35.91	789.36	6.29	0.51	54.93
	SD	0.26	0.5	1.2	0.82	0.32	0.66	86.25	0.27	0.06	1.91
IP 24 h	*N*	6	6	6	6		6	6	6	6	6
	*X*	8.69	15.78	43.48	49.85	18.13	36.38	642.33	6.73	0.43	52.0
	SD	0.21	0.50	1.08	0.80	0.39	0.37	109.31	0.26	0.07	52.54
	*T*	0.189	0.221	0.435	0.123	0.911	0.122	0.005**	0.003**	0.031*	0.012*
IP 72 h	*N*	6	6	6	6	6	6	6	6	6	6
	*X*	7.51	13.60	38.18	51.05	18.18	35.60	600.33	7.20	0.43	51.53
	SD	0.54	0.86	2.70	1.11	0.45	0.30	153.00	0.85	0.09	1.02
	*T*	0.0000***	0.0000***	0.0000***	0.224	0.702	0.281	0.002**	0.002**	0.037*	0.0007***
IP 120 h	*N*	9	9	9	9	9	9	9	9	9	9
	*X*	7.40	13.53	37.27	50.36	18.30	36.33	803.33	7.24	0.58	16.26
	SD	0.58	0.87	2.62	0.85	0.37	0.56	70.84	0.13	0.05	0.25
	*T*	0.0000***	0.0000***	0.0000***	0.703	0.218	0.130	0.689	0.0000***	0.06	0.0000***
IPD 24 h	*N*	6	6	6	6	6	6	6	6	6	6
	*X*	6.71	12.43	34.25	50.58	18.37	36.32	746.83	6.32	0.47	17.18
	SD	0.35	0.60	1.57	0.70	0.19	0.26	41.47	0.13	0.03	0.34
	*T*	0.0000***	0.0000***	0.0000***	0.122	0.218	0.228	0.053	0.006**	0.213	0.0000***
IPD 72 h	*N*	6	6	6	6	6	6	6	6	6	6
	*X*	6.52	12.07	33.43	50.62	18.23	36.20	623.00	6.43	0.40	17.33
	SD	0.36	0.62	1.66	1.29	0.60	0.36	74.84	0.11	0.05	0.18
	*T*	0.004**	0.005**	0.004**	0.546	0.874	0.010*	0.751	0.053	0.533	0.0000***
IPD 120 h	*N*	7	7	7	7	7	7	7	7	7	7
	*X*	6.92	12.53	34.67	50.20	18.10	36.10	671.57	6.96	0.46	16.27
	SD	0.76	1.09	2.98	1.42	0.54	0.49	192.77	0.25	0.13	0.72
	*T*	0.172	0.058	0.085	0.789	0.393	0.399	0.077	0.010*	0.028*	0.0000***

Explanation of symbols: number of animals (*N*), arithmetic means of parameters (*X*), standard deviation (SD)

Statistical significance divided into: *—0.05 ≥ *P* > 0.01); **—0.01 ≥ *P* > 0.001); ***—0.001 ≥ *P*; *NS* not significant

**Table 2:** Correlation Coefficients *r* between Hematological Parameters and Inflammation Duration

*t* [h]	RBC	HGB	HCT	MCV	MCH	MCHC
IP	*r =* −**0.7250**	*r =* −**0.7316**	*r =* −**0.7395**	*r =* 0.2122	*r =* 0.1840	*r =* −0.0388
	*P* = **0.001**	*P* = **0.001**	*P =* **0.000**	*P =* 0.398	*P =* 0.465	*P* = 0.879
IPD	*r* = 0.1691	*r* = 0.0538	*r* = 0.0838	*r* = −0.1420	*r* = −0.2445	*r =* −0.2495
	*P =* 0.502	*P =* 0.832	*P =* 0.741	*P =* 0.574	*P =* 0.328	*P =* 0.318

Statistically significant dependencies are in bold

#### Hemoglobin (HGB)

The HGB concentration in the IP group at the 24th hour of pleuritis is almost the same as in the control group (Fig. 3b). The second and third blood measurements have shown a significant drop of HGB in the IP group in comparison to the control group (Table 1). TCDD administration causes the HGB level to decrease in the IPD group compared to the IP group between 1 and 120th hour since carrageenan injection. The difference of HGB value between the IP and the IPD group is statistically significant at the 24th and 72nd hour of pleuritis. The correlation of this parameter *versus* time in the IP group is negative, very high, and statistically significant (*P* = 0.001). On the contrary, in the IPD group, statistically insignificant dim positive correlation has been calculated (Fig. 4b, Table 2).

#### Hematocrit (HCT)

The HCT changes occur in a similar way as RBC alteration in both the IP and IPD groups in comparison to the control group. First, at the 24th hour of pleuritis, the HCT value insignificantly increases in the IP group compared to the control group, and then continuously drops (Fig. 3c). The HCT decrease is statistically significant between the 72nd and 120th hour of the inflammation in the IP group. Similarly, TCDD administration into the IPD group causes HCT values to lower in the IPD group than in the IP group at all three measurement points, and the differences of HCT values between the IP and the IPD group are statistically significant at the 24th and 72nd hour of pleuritis (Table 1). The correlation of HCT *versus* time in the IP group is negative, very high, and statistically significant (*P* = 0.000), whereas the correlation in the IPD group is dim positive and statistically insignificant (Fig. 4c, Table 2).

#### Mean Corpuscular Volume of Hemoglobin (MCV)

**Fig. 5:**
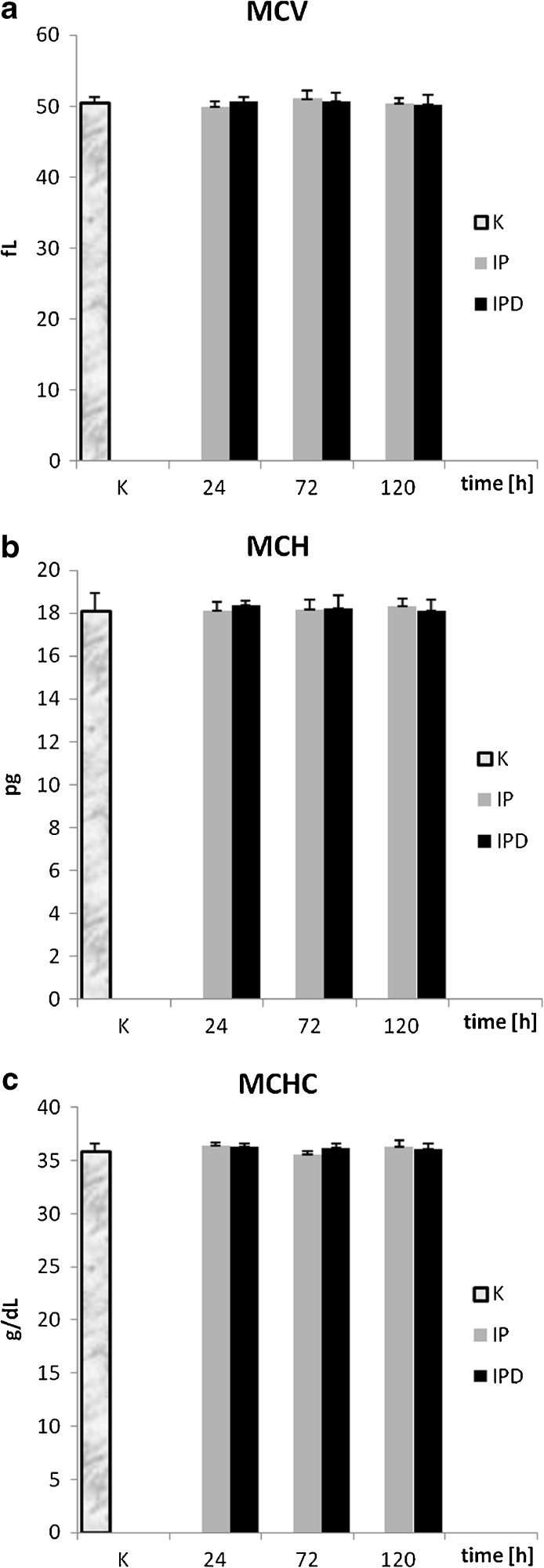
**a** The influence of TCDD on mean corpuscular volume during the experimentally induced pleuritis in rats. **b** The influence of TCDD on mean hemoglobin parameter during the experimentally induced pleuritis in rats. **c** The influence of TCDD on mean corpuscular hemoglobin concentration during the experimentally induced pleuritis in rats.

MCV values in each blood measurement are on similar level in the control, IP, and IPD groups (Fig. 5a, Table 1). Neither the inflammation induction nor TCDD application influences significantly the MCV values. For this parameter, the correlation *versus* time in both the IP and IPD groups is weak and statistically insignificant; however, in the IP group, it is positive, and in the IPD group, it is negative (Table 2).

#### Mean Corpuscular Hemoglobin Parameter (MCH)

No changes of MCH values are observed in the IP and IPD groups in comparison to the control group in each blood measurement (Fig. 5b, Table 1). The inflammation state and TCDD treatment do not influence MCH values. The correlation of MCH *versus* time in the IP group is weak positive and statistically insignificant (Table 2), whereas the correlation in the IPD group is weak negative and statistically insignificant.

#### Mean Corpuscular Hemoglobin Concentration (MCHC)

MCHC values, similar to MCV and MCH values, are on a similar level in the control, IP, and IPD groups in each blood measurement (Fig. 4c, Table 1). Neither the inflammation induction nor TCDD application has a significant influence on MCV values. The correlation of MCHC in relation to time in both the IP and IPD groups is statistically insignificant, negative, and very weak for the IPD group and dim for the IP group (Table 2).

#### Platelets (PLT)

**Fig. 6:**
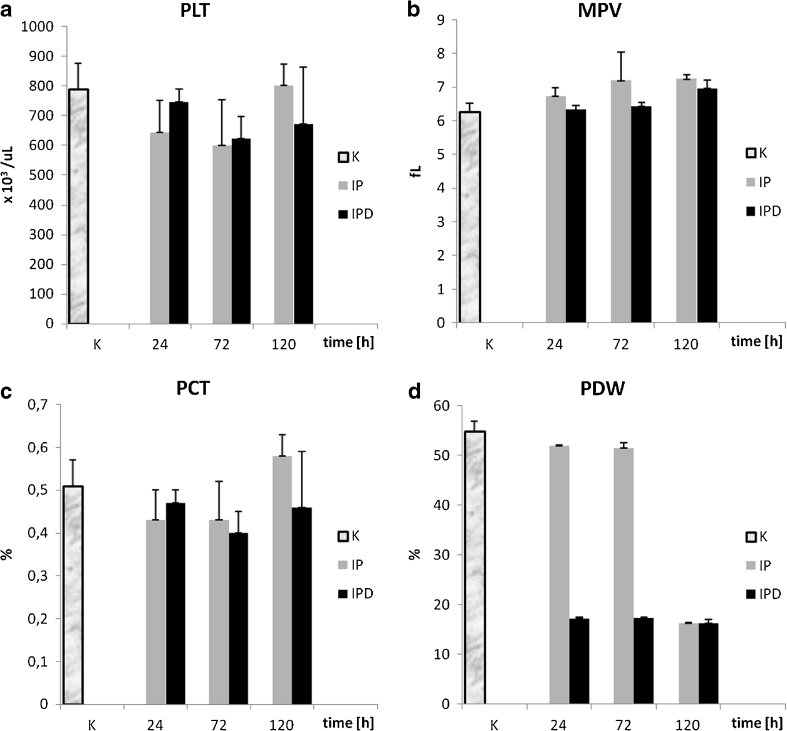
**a** The influence of TCDD on the number of thrombocytes during the experimentally induced pleuritis in rats. **b** The influence of TCDD on mean platelet volume during the experimentally induced pleuritis in rats. **c** The influence of TCDD on thrombocrit parameter during the experimentally induced pleuritis in rats. **d** The influence of TCDD on platelet distribution width during the experimentally induced pleuritis in rats.

**Fig. 7:**
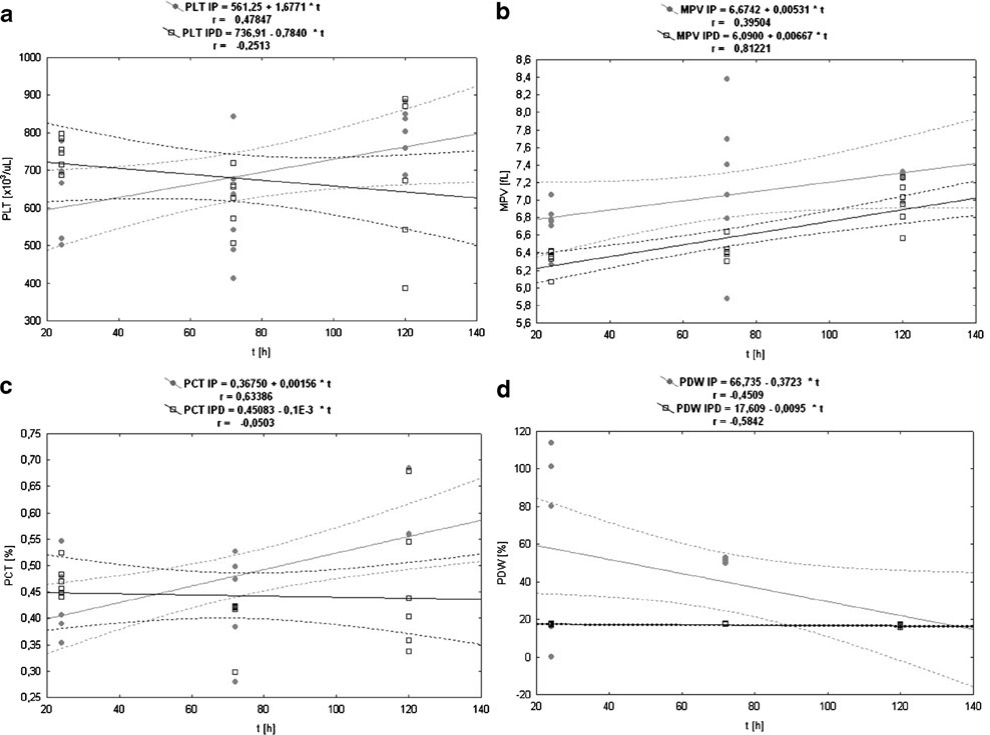
**a** The linear regression of the influence of experimentally induced pleuritis (IP) and dioxin exposition (IPD) on the platelet (PLT) parameter in rats. **b** The linear regression of the influence of experimentally induced pleuritis (IP) and dioxin exposition (IPD) on the mean platelet volume (MPV) parameter in rats. **c** The linear regression of the influence of experimentally induced pleuritis (IP) and dioxin exposition (IPD) on the thrombocrit (PCT) parameter in rats. **d** The linear regression of the influence of experimentally induced pleuritis (IP) and dioxin exposition (IPD) on the platelet distribution width (PDW) parameter in rats.

The PLT level is the highest in the control group in comparison to PLT values from the other groups in each blood measurement (Fig. 6a). Pleuritis induction causes a statistically significant drop of PLT number in the IP group at the 24th and 72nd hour compared to the control group (Table 1). The third blood measurement at the 120th hour of the experiment indicates a significant increase of PLT number in the IP group compared to the first and second PLT measurements at the 24th and 72nd hour in this group. TCDD treatment causes a statistically significant increase of PLT value in the IPD group at the 24th hour in relation to the IP group. The PLT numbers at the 72nd hour of the inflammation are on a similar level in the IP and IPD groups. The third blood measurement at the 120th hour of the experiment shows no changes of PLT number in the IPD group in comparison to PLT values in this group at the 72nd hour of inflammation. In the IP group, statistically significant (*P* = 0.045) average positive correlation has been calculated (Table 3). In contrast, the correlation in the IPD group is negative, weak, and statistically insignificant (Fig. 7a).

**Table 3:** Correlation Coefficients *r* between Hematological Parameters and Inflammation Duration

*t* [h]	PLT	MPV	PCT	PDW
IP	***r =*** **0.4785**	*r* = 0.3950	***r*** **= 0.6339**	*r* = −0.4509
	***P*** **= 0.045**	*P* = 0.105	***P*** **= 0.005**	*P* = 0.060
IPD	*r* = −0.2513	***r*** **= 0.8122**	*r* = −0.0503	***r*** **= −0.5842**
	*P* = 0.315	***P*** **= 0.000**	*P* = 0.843	***P*** **= 0.011**

Statistically significant dependencies are in bold

#### Mean Platelet Volume (MPV)

The MPV value is the lowest in the control group in comparison to MPV values from the other groups in each blood measurement (Fig. 6b). Pleuritis initiation causes continuous increase of MPV value in both IP and IPD groups compared to the control group (Table 1). MPV differences between the control and IP group are statistically significant in every MPV measurement. TCDD application is responsible for MPV decreases in the IPD group relative to the IP group. However, the difference in MPV values between the IP and the IPD group is statistically significant only at the 24th hour of pleuritis. For this parameter, the correlation *versus* time in both the IP and IPD groups is positive (Table 3, Fig. 7b); however, in the IP group, it is average and statistically insignificant, and in the IPD group, it is very high and statistically significant (*P* = 0.000).

#### Thrombocrit (PCT)

PCT values are lower in the IP group than in the control group at the 24th and 72nd hour of pleuritis, and these differences are statistically significant (Fig. 6c). The third PCT measurement at the 120th hour of the inflammation indicates similar PCT values in the control and the IP group. TCDD administration has no significant influence on the PCT values, and they are on a similar level in the IPD and IP groups during 72 h of the experiment. The third blood measurement at the 120th hour shows statistically significant lower PCT value in the IPD group than in both the IP and the control groups. In the IP group, a statistically significant (*P* = 0.005) high positive correlation has been calculated (Table 3, Fig. 7c). In contrast, the correlation in the IPD group is negative, dim, and statistically insignificant.

#### Platelet Distribution Width (PDW)

PDW values are on a similar level in the IP and control groups at the 24th and 72nd hour of the experiment (Fig. 6d). The third blood measurement at the 120th hour of the inflammation indicates a drastic PDW drop in the IP (Table 1). PDW value at the 120th hour of pleuritis in the IP group reaches 30 % of the value of the control group. TCDD administration on the third week before carrageenan application causes a statistically significant PDW drop, which is proven by all three blood measurements. The first PDW measurement in the 24th hour of the inflammation and its value are maintained during the entire duration of the experiment. PDW value in the IPD group in each blood measurement point is similar to PDW level of the IP group at the 120th hour of pleuritis and reaches 30 % of the value of the control group. The differences between the IP and the IPD group are statistically significant at the 24th, 72nd, and 120th hour of pleuritis. The correlation of this parameter in relation to time in both the IP and IPD groups is negative; however, in the IP group, it is average and insignificant, and in the IPD group, it is statistically significant (*P* = 0.011) and high (Fig. 7d, Table 3).

#### Leukocytes (WBC)

**Fig. 8:**
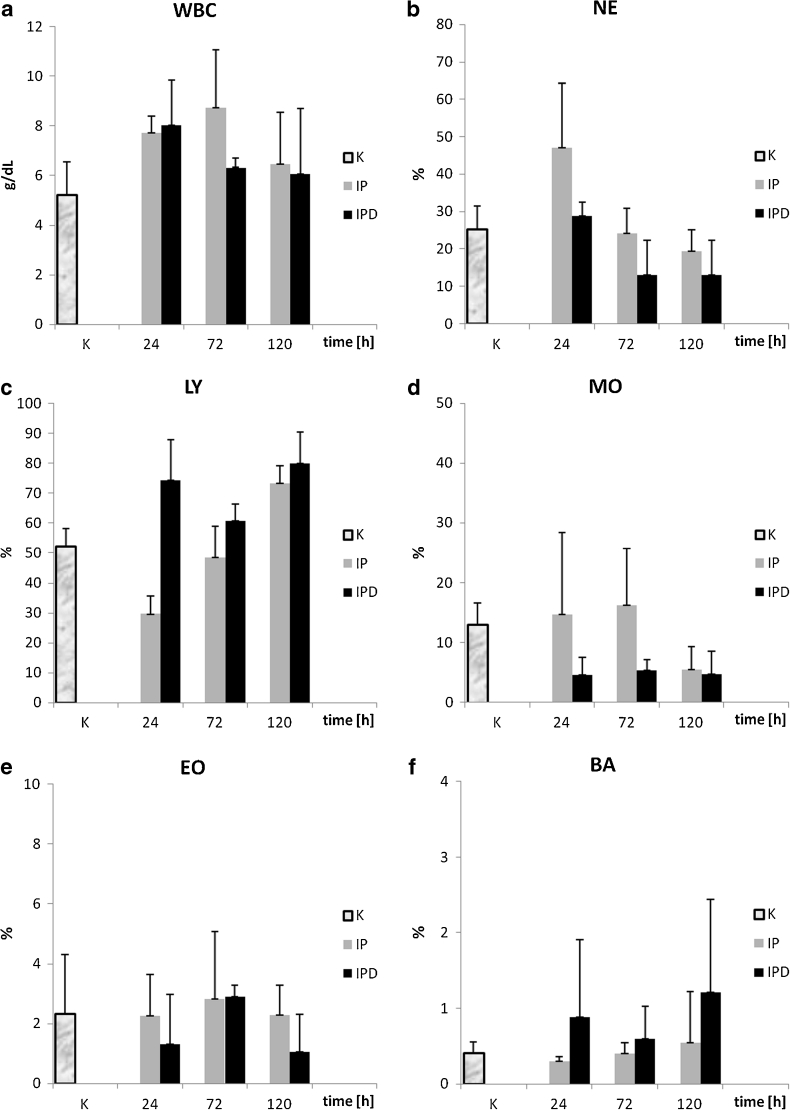
**a** The influence of TCDD on white blood cell concentration during the experimentally induced pleuritis in rats. **b** The influence of TCDD on the number of neutrophils during the experimentally induced pleuritis in rats. **c** The influence of TCDD on the number of lymphocytes during the experimentally induced pleuritis in rats. **d** The influence of TCDD on the number of monocytes during the experimentally induced pleuritis in rats. **e** The influence of TCDD on the number of eosinophiles during the experimentally induced pleuritis in rats. **f** The influence of TCDD on the number of basophiles during the experimentally induced pleuritis in rats.

The WBC level is the lowest in the control group compared to WBC values from the other groups in each blood measurement (Fig. 8a). Furthermore, WBC differences between the control and IP group are statistically significant during each blood measurement (Table 4). Pleuritis initiation causes a continuous increase of WBC concentration in both the IP and IPD groups compared to the control group, which is proven by all three blood measurements. The highest WBC value appears at the 72nd hour of the inflammation in the IP group compared to other blood measurements from the control, IP, and IPD groups. TCDD application causes a gentle WBC increase at the 24th hour of the inflammation (IPD group *versus* IP group). The second blood measurement points to a drastic WBC decrease in the IPD group compared to the IP group at the 72nd hour of pleuritis. This difference is statistically significant. WBC level in the IPD group receives a similar value as in the IP group at the 120th hour of the inflammation. The correlation of absolute value of this parameter in relation to time in both the IP and IPD groups is statistically insignificant, negative, and average for the IPD group and weak for the IP group (Table 5).

**Table 4:** Values of Leukocyte Blood Parameters Received at the 24th, 72nd, and 120th Hour of the Experiment

		WBC (×10^3^/μL)	NE (%)	LY (%)	MO (%)	EO (%)	BA (%)
K	*N*	14	14	12	12	12	12
	*X*	5.22	25.21	52.36	12.94	2.33	0.41
	SD	1.33	6.28	5.81	3.61	1.99	0.14
IP 24 h	*N*	6	6	6	6	6	6
	*X*	7.73	47.03	29.62	14.62	2.27	0.30
	SD	0.67	17.27	5.91	13.72	1.38	0.06
	*T*	0.0004***	0.0005***	0.0000***	0.689	0.950	0.089
IP 72 h	*N*	6	6	6	6	6	6
	*X*	8.74	24.12	48.45	16.18	2.82	0.40
	SD	2.33	6.68	10.54	9.47	2.27	0.14
	*T*	0.0004***	0.729	0.320	0.302	0.643	0.906
IP 120 h	*N*	9	9	9	9	9	9
	*X*	6.47	19.36	73.43	5.39	2.28	0.54
	SD	2.09	5.68	5.83	3.89	1.00	0.68
	*T*	0.093	0.015*	0.0000***	0.0002***	0.949	0.506
IPD 24 h	*N*	6	6	6	6	6	6
	*X*	8.03	12.48	74.40	4.60	1.32	0.88
	SD	1.82	11.67	13.52	2.85	1.65	1.03
	*T*	0.711	0.002**	0.0000***	0.110	0.304	0.196
IPD 72 h	*N*	6	6	6	6	6	6
	*X*	6.32	28.70	60.68	5.35	2.90	0.60
	SD	0.39	3.70	5.72	1.80	0.40	0.43
	*T*	0.031*	0.172	0.032*	0.020*	0.931	0.306
IPD 120 h	*N*	7	7	7	7	7	7
	*X*	6.06	12.99	80.07	4.67	1.06	1.21
	SD	2.65	9.28	10.27	3.86	1.26	1.23
	*T*	0.734	0.174	0.123	0.719	0.049*	0.186

Explanation of symbols: number of animals (*N*), arithmetic means of parameters (*X*), standard deviation (SD)

Statistical significance divided into: *—0.05 ≥ *P* > 0.01); **—0.01 ≥ *P* > 0.001; ***—0.001 ≥ *P*; *NS* not significant

**Table 5:** Correlation Coefficients *r* between Hematological Parameters and Inflammation Duration for Absolute Value of Parameters [×10^3^/μL]

*t* [h]	WBC	NE	LY	MO	EO	BA
IP	*r* = −0.2671	***r*** **= −0.8216**	***r*** **= 0.6347**	*r* = −0.4001	*r* = −0.1269	***r*** **= −0.8404**
	*P* = 0.284	***P*** **= 0.000**	***P*** **= 0.005**	*P* = 0.100	*P* = 0.616	***P*** **= 0.000**
IPD	*r* = −0.4177	*r* = −0.1982	*r* = −0.1933	*r* *=* −0.0691	*r* = −0.1550	*r* = −0.0919
	*P* = 0.085	*P* = 0.430	*P* = 0.442	*P* = 0.785	*P* = 0.539	*P* = 0.717

Statistically significant dependencies are in bold

#### Neutrophils (NE)

**Fig. 9:**
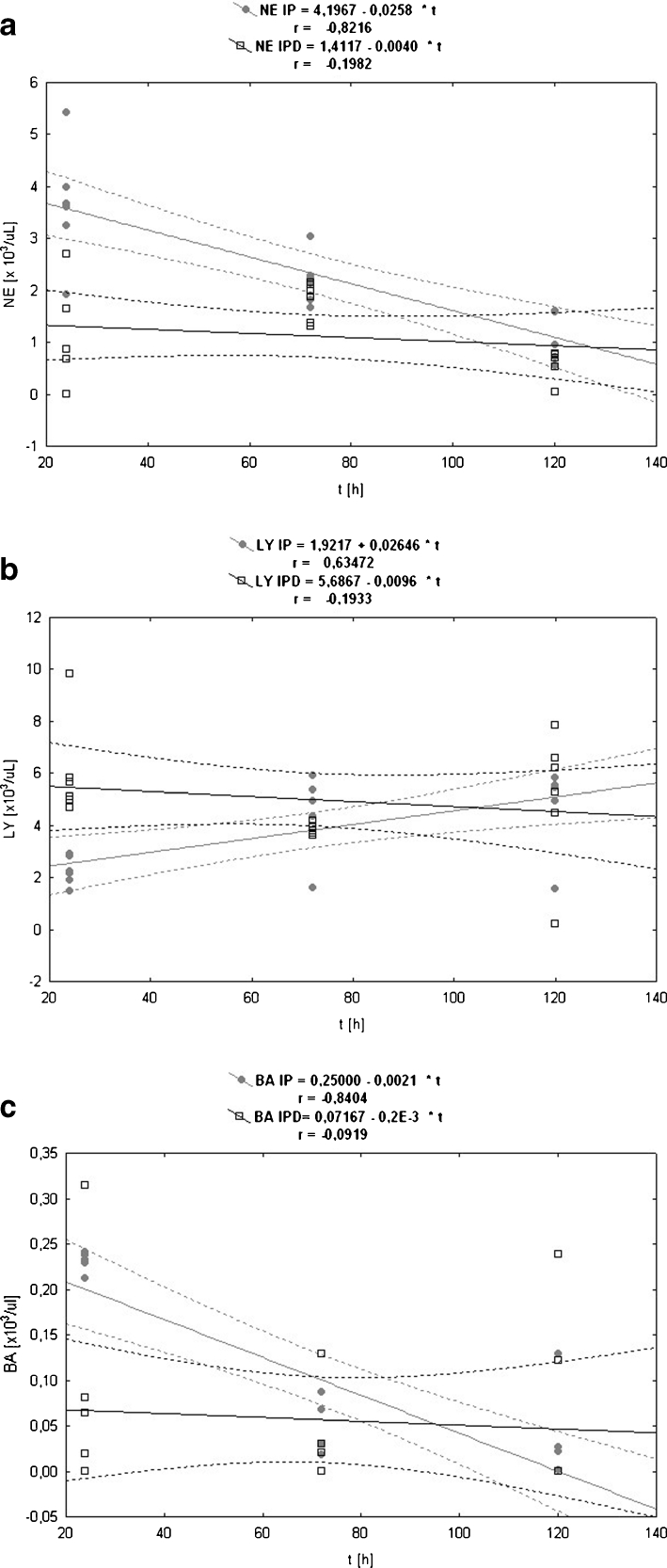
**a** The linear regression of the influence of experimentally induced pleuritis (IP) and dioxin exposition (IPD) on the neutrophil (NE) parameter in rats. **b** The linear regression of the influence of experimentally induced pleuritis (IP) and dioxin exposition (IPD) on the lymphocyte (LY) parameter in rats. **c** The linear regression of the influence of experimentally induced pleuritis (IP) and dioxin exposition (IPD) on the basophile (BA) parameter in rats.

The changes of NE levels in the control, IP, and IPD groups have different profiles than WBC changes (Fig. 8b). Pleuritis initiation causes a drastic increase of NE number in the IP group at the 24th hour of the inflammation compared to the control group (Table 4). The first blood measurement at the 24th hour of the inflammation points that TCDD application causes the NE drop in the IPD group and its level is similar to the one in the control group. Moreover, the NE difference between the IP and the control group is statistically significant at the 24th hour of the inflammation. Subsequent blood measurements at the 72nd and 120th hour point to continuous NE decrease in both the IP and IPD groups. The NE value is lower in the IPD group than in the IP group in every measurement point, and these differences are statistically significant. For the absolute value of this parameter, the correlation *versus* time in both the IP and the IPD group is negative; however, in the IP group, it is very high and statistically significant (*P* = 0.000), and in IPD group, it is weak and statistically insignificant (Table 5). For relative values of NE, the correlation in the IP group remains unchanged, but in the IPD group, it becomes dim positive and remains insignificant (Fig. 9a).

#### Lymphocytes (LY)

The pleuritis initiation in the IP group causes the LY drop at the 24th hour of the inflammation compared to the control group (Fig. 8c). Moreover, TCDD application is responsible for drastic LY increase in the IPD group in relation to the IP group at the 24th hour of pleuritis (Table 4). This LY difference is statistically significant and the highest in comparison to other LY comparisons between the IP and IPD groups. The LY level rises continuously in the IP group at the 24th, 72nd, and 120th hour of pleuritis and reaches the highest value at the 120th hour in comparison to other blood measurements from every group. In the IP group, a statistically significant (*P* ≤ 0.005) positive correlation for both absolute and relative values has been calculated. There occurred a high correlation for the absolute value and almost total correlation for the relative value (Fig. 9b, Table 5). In the IPD group, the correlation is weak, insignificant, and negative for absolute values and positive for relative values.

#### Monocytes (MO)

Pleuritis initiation causes the MO increase during first 72 h of the inflammation in the IP group in relation to the control group (Fig. 8d). The third blood measurement points to a drastic MO drop at the 120th hour of pleuritis in the IP group in comparison to other MO measurements in this group—its value reaches 50 % of the MO value of the control group at the 120th hour of the inflammation (Table 4). Moreover, the difference of MO value between the IP and the control group is statistically significant only at the 120th hour of pleuritis. Furthermore, TCDD application causes a statistically significant decrease of MO value in the IPD group in every measurement point in relation to proper MO level from the IP group. TCDD presence in the IPD group is probably responsible for the statistically significantly lower MO levels in IPD group according to both the IP and control groups for the whole duration of the inflammatory reaction. The correlation of the absolute value of this parameter in relation to time in both the IP and IPD groups is statistically insignificant, negative, and dim for the IPD group and average for the IP group. For relative values of MO, the correlation in the IP group remains unchanged, but in the IPD group, it becomes dim positive and remains insignificant.

#### Eosinophiles (EO)

Pleuritis initiation as well as earlier TCDD injection does not significantly influence EO levels. Differences between the control, IP, and IPD groups are not statistically significant during the whole time of the inflammation (Fig. 8e). For this parameter, the correlation *versus* time for all parameters is statistically insignificant (Table 4). A weak negative correlation for absolute values has been calculated. For relative values of this parameter, correlation is dim in both the IP and IPD groups; however, it is positive in the IP group and negative in the IPD group.

#### Basophiles (BA)

BA levels, similar to EO levels, are not significantly changed after pleuritis initiation (Fig. 8f). TCDD application in the 3rd week before carrageenan administration does not influence BA values. Differences in BA concentration in the control, IP, and IPD groups are not statistically significant (Table 4). The correlation of this parameter in relation to time for absolute values of this parameter is negative (Fig. 9c, Table 5); however, it is significant and very high in the IP group and statistically insignificant and dim in the IPD group. In contrast, for relative values, the correlation is weak positive and statistically insignificant in both the IP and IPD groups.

## DISCUSSION

The inflammatory reaction contributes to a change in the values of erythrocyte indices. Probably, it is a result of intravascular hemolysis [4, 38]. Similar changes are observed in rheumatoid arthritis for which the reduction of the MCHC and the mean corpuscular volume (MCV) are characteristic features. In this situation, low iron concentration in serum and accelerated fibrinogen concentration have been shown [18, 28]. The complement system is activated in the coagulation process and kinin concentration rises [39, 40]. The role of fibrinogen in the inflammation as a protein of an acute phase is connected with clot formation in inflammatory focuses which detain mainly erythrocytes. This process causes a decrease in the amount of fibrinogen and erythrocytes in blood (depending on the area of the inflammation) and occurrence of anemia with the iron drop in serum [1, 3, 41]. The erythrocyte decrease during the inflammation can also be caused by DIC syndrome, pH drop in inflammatory focuses, or by changes of various metabolites such as increase of urea, lactic acid, or proteolytic enzyme concentrations [3]. The activation of compounds of the complement system is also responsible for erythrocyte resistance [3]. The current study suggests that the disposable TCDD dose administered to rats 3 weeks before the pleuritis induction by using carrageenan significantly influences the decrease of RBC, HGB, HCT, and MPV. The obtained results may be explained by the notable TCDD influence on the erythropoiesis process and uncompensated erythrocyte decline, which can also have an effect on the long-lasting inflammatory reaction [42, 43]. According to other studies, the decrease of hemoglobin concentration and erythrocyte number is noticed after application of higher doses of TCDD [44–46]. The number of erythrocytes decreases and is connected with the decrease of HGB and HTC, shown in the current study, which can be the result of the resistance loss in an inflammation focus caused by chemical compounds, complement proteins such as C3 or C4 as well as by the DIC syndrome proven by histopathological studies and fibrinogen or platelet losses [3]. Furthermore, this effect can be explicated by the interaction of TCDD with DNA in cells which are able to divide [47, 48]. Although changes of total platelet amount (PLT) in rats with acute pleuritis being under TCDD are not observed, the alteration of the rest of the platelet parameters indicates the regeneration process of platelets. The presence of TCDD in the inflammatory reaction does not significantly influence white blood cells, particularly the number of general leukocytes compared to the control group (without the inflammation). The essential changes occur in the ratio of the number of lymphocytes to the number of neutrophils. The decrease in the amount of monocytes and eosinophiles is also significant [21, 22]. These changes in animals with pleuritis being under TCDD indicate a distinct type of leukocytic response with the predominance of lymphocytes in relation to animals treated only with carrageenan. The results obtained by other authors do not indicate TCDD influence on lymphocytic cultures [49, 50]. We would like to underline that the rats used in the experiments were 8 weeks old and had a developed thymus. According to other studies, application of TCDD dose seven times higher causes thymus, spleen, or lymphatic gland atrophy [51, 52]. Our studies also show the same effects of dioxin, despite using the seven times lower dose of TCDD [44, 45, 53]. Different TCDD doses are probably a cause of a distinct white blood cell picture in rats with experimentally induced pleuritis being under the effect of TCDD received in current and recent studies. According to Vos *et al.* [54, 55], low TCDD doses (about 0.2 μg per 1 kg of body weight) influence the humoral response displaying itself through the increase of α- and β-globulin levels and reduction of the ability for antibody synthesis in mice plasma. The dioxin’s affinity to DNA molecules suggests that dioxins could be responsible for the immunity decrease [45, 54, 55].

The considerable influence of dioxin on the inflammation process is also proven by the statistical analysis carried out on the above results. Current studies indicate that a statistically significant very high correlation occurs in the erythrocyte system, and inflammation significantly affects the changes in RGB, HGB, and HCT parameters. Interestingly, the presence of TCDD during induced pleuritis reverses the relationships between these parameters, making them dim or weakly positive and insignificant. The reverse of the trend of correlation in the IP and IPD groups has been observed for other red blood cell parameters; however, these changes were insignificant and the correlation was dim or weak. Changes in thyroid hormone economy and the adrenal cortex caused by TCDD administration may significantly influence certain metabolic and hematological indices (erythrocyte and leukocyte) [56]. Disorders of erythropoiesis and hemoglobin synthesis may be the result of the described endocrine disorders causing dysfunction of the ovaries and thus contributing to the impairment of estrogen [57, 58].

Moreover, the influence of TCDD on the inflammation process is very noticeable for all platelet factors. Importantly, two types of dependencies could be distinguished in this group. The first occurs for PLT and PCT, where statistically significant average and high positive correlation in induced pleuritis turns into insignificant dim or weak negative correlation. Furthermore, the second dependency observed in MPV and PDW parameters have shown that the trend of correlation remains unchanged, and a higher value of correlation and statistically significant changes occur in the IDP group; however, correlation *versus* time is positive for MPV and negative for PDW parameters. For absolute values of leukocyte parameters, except lymphocyte, a negative correlation occurs for both the IP and IPD groups; however, the correlation becomes stronger for WBC and remains unchanged for eosinophiles. It is noteworthy that a weaker correlation has been shown for monocytes and also for neutrophils and basophiles, in which the changes of parameter value during inflammation are significant. In contrast, results for lymphocytes are positively correlated in induced pleuritis and the change during the inflammation process is significant. This high positive dependency changes into weak negative after dioxin exposition. The results indicate a change in response to an inflammatory reaction from neutrophilic in the IP group to leukocytic in the IPD group. This observation has some diagnostic significance and may help to avoid misinterpretation of the nature of the inflammation in humans and animals in which the inflammation is caused by bacteria or viruses [59]. Summarizing the observations, it is concluded that TCDD destabilized the erythrocyte, platelet, and leukocyte systems’ response in comparison to the group of controlled inflammation, in which correct cellular response can be proven.

## CONCLUSIONS

The results of this study have shown that dioxins contribute to a change of the character of the inflammatory reaction, especially in both erythrocyte and leukocyte systems.

1. TCDD administration 3 weeks before pleuritis initiation causes a statistically significant decrease of RBC, HGB, and HCT values in every three measurement points at the 24th, 72nd, and 120th hour of the inflammation. Consequently, the significant loss of the numbers of erythrocytes in blood explains the deficiency of changes in other erythrocyte indices. The erythrocyte loss is connected with erythrocyte resistance caused by the inflammation accelerated by the presence of TCDD. However, the decrease of HGB and HCT, which is the cause of reduction in the DIC syndrome, does not lead to erythropoiesis because other parameters—MCV, MCH, MCHC—are not changed.
2. On the other hand, the presence of TCDD is responsible for the increase of PLT at the 24th hour of pleuritis and the decrease of both MPV and PDW values during the whole time of the inflammation. Dioxins influence platelet number—their loss in the first stage of the inflammation is caused by their excessive reduction. Furthermore, in the later stage of the inflammation, TCDD has influence on thrombopoiesis—they lead to the creation of bigger forms.
3. The action of TCDD accelerates the lymphocytic response in comparison to reactions carried out on experimental animals without the application of TCDD. Single TCDD administration 3 weeks before pleuritis initiation causes the increase of LY and BA values during the whole time of the inflammation and WBC rise at the 24th hour of the inflammation in the IPD group in relation to the IP group. On the other hand, the NE and MO amounts are significantly lower in the group of rats with pleuritis under TCDD in comparison to animals with controlled inflammation, without TCDD application. Studies have shown that dioxins change the immunity response characteristic during inflammatory reaction. They accelerate lymphocyte interaction and weaken neutrophil action.

## Abbreviations

BA: Basophiles
b.w.: Body weight
COX-2: Cyclooxygenase enzyme II
DIC: Disseminated intravascular coagulation
EO: Eosinophiles
HCT: Hematocrit
HGB: Hemoglobin
LY: Lymphocytes
MCH: Mean corpuscular hemoglobin
MCHC: Mean corpuscular hemoglobin concentration
MCV: Mean corpuscular volume
MO: Monocytes
MPV: Mean platelet volume
NE: Neutrophils
PCT: Thrombocrit
PDW: Platelet distribution width
PLT: Platelets
RBC: Erythrocytes
RDW: Red blood cell distribution width
TCDD: 2,3,7,8-Tetrachlorodibenzo-*p*-dioxin
WBC: Leukocytes
